# Temporal trajectories of accompanying comorbidities in patients with type 2 diabetes: a Korean nationwide observational study

**DOI:** 10.1038/s41598-020-62482-1

**Published:** 2020-03-26

**Authors:** Eugene Jeong, Namgi Park, Yujeong Kim, Ja Young Jeon, Wou Young Chung, Dukyong Yoon

**Affiliations:** 1 Department of Biomedical Informatics, Vanderbilt University School of Medicine, Nashville, TN USA; 2 Department of Biomedical Informatics, Ajou University School of Medicine, Suwon, Gyeonggi-do Republic of Korea; 3 Department of Biomedical Sciences, Ajou University Graduate School of Medicine, Suwon, Gyeonggi-do Republic of Korea; 4 Department of Endocrinology and Metabolism, Ajou University School of Medicine, Suwon, Gyeonggi-do Republic of Korea; 5 Department of Pulmonology and Critical Care Medicine, Ajou University School of Medicine, Suwon, Gyeonggi-do Republic of Korea

**Keywords:** Endocrinology, Endocrine system and metabolic diseases, Diabetes, Diabetes complications, Type 2 diabetes

## Abstract

Type 2 diabetes mellitus is a major concern globally and well known for increasing risk of complications. However, diabetes complications often remain undiagnosed and untreated in a large number of high-risk patients. In this study based on claims data collected in South Korea, we aimed to explore the diagnostic progression and sex- and age-related differences among patients with type 2 diabetes using time-considered patterns of the incidence of comorbidities that evolved after a diagnosis of type 2 diabetes. This study compared 164,593 patients who met the full criteria for type 2 diabetes with age group-, sex-, encounter type-, and diagnosis date-matched controls who had not been diagnosed with type 2 diabetes. We identified 76,423 significant trajectories of four diagnoses from the dataset. The top 30 trajectories with the highest average relative risks comprised microvascular, macrovascular, and miscellaneous complications. Compared with the trajectories of male groups, those of female groups included relatively fewer second-order nodes and contained hubs. Moreover, the trajectories of male groups contained diagnoses belonging to various categories. Our trajectories provide additional information about sex- and age-related differences in the risks of complications and identifying sequential relationships between type 2 diabetes and potentially complications.

## Introduction

Diabetes mellitus is among the most prevalent disorders worldwide, affecting approximately 451 million adults (age: 18–99 years) in 2017 according to the International Diabetes Federation (IDF)^1–3^. The prevalence of diabetes mellitus has also increased exponentially in recent decades and is expected to reach 693 million cases within 25 years^4^. Consequently, the healthcare costs associated with diabetes constitute a growing burden on financial and health systems around the world. In 2017, the estimated total global healthcare expenditure regarding on diabetes mellitus was USD 850 billion for adults, and this value is expected to increase to USD 958 billion by 2045^4^. A considerable proportion of these associated costs of arise from the treatment of various complications associated with the progression of diabetes mellitus. Therefore, the early diagnosis and close monitoring of this disease would necessarily minimize the associated healthcare costs, as well as the risk of complications. However, approximately 193 million affected patients remain undiagnosed before developing long-term complications caused by uncontrolled chronic hyperglycemia^4^.

Currently, type 2 diabetes accounts for more than 90% of all newly diagnosed cases of diabetes mellitus in adults^5,6^. This condition is associated with an increased risk of various complications, which can be categorized into three major groups: microvascular, macrovascular, and miscellaneous^7^. Microvascular complications, which affect small vessels, are induced by several mechanisms related to chronic hyperglycemia, including the production of advanced glycation end products [AGEs], a proinflammatory microenvironment, and the induction of oxidative stress^8,9^. These mechanisms can lead to diabetic nephropathy, neuropathy, and retinopathy^10^. Macrovascular complications, which affect the large vessels of the body, are usually caused by atherosclerosis and may lead to stroke, acute myocardial infarction, or vessel blockage in the legs (i.e., peripheral vascular disease). A recent review suggested that although microvascular complications distinctly precede macrovascular complications, both progress simultaneously on a continuum^11^.

Most type 2 diabetes-related complications progress over a period of years. Therefore, it would be useful to know the expected clinical trajectories as these data would not only guide the decisions regarding and delivery of early preventive care, but may also help physicians to explain the course of type 2 diabetes and warn their patients about complications. Moreover, the prevalence of type 2 diabetes-related complications is known to differ with respect to age, sex, ethnicity and duration of diabetes^12–19^. Therefore, an analysis of the factors affecting the trajectories of complications could provide new insights into patient management, prevention, or treatment. However, most published studies on the complications of type 2 diabetes were limited to one or a few complications and applied large-scale approaches without time considerations. For example, Jeong *et al*. developed a diagnostic progression network based on claims data with the aim of determining the global patterns of diagnosis in South Korea^20^. However, this network provided only information about the associations between diagnosis pairs, rather than the sequential trajectories. Furthermore, Jensen *et al*. used electronic patient records from a Danish population to suggest temporal trajectory clusters of various diseases, including diabetes, and thus predict disease evolution over time^21^. However, these trajectories were only evaluated using full population data, rather than data stratified by different combinations of sex and age.

In this study, we aimed to construct time-dependent type 2 diabetes trajectories based on population-wide claim data. These trajectories would be expected to reveal time-critical associations between type 2 diabetes and other diseases and identify differences in the patterns of progression among various age and sex groups. To overcome the insufficient definition of a diagnosis simply as a direct complication of type 2 diabetes according to sequential patterns from claims data, we used the term “accompanying comorbidity” rather than “complication” to encompass all possible relationships with type 2 diabetes.

## Results

### Clinical and demographic characteristics of participants in the case-control study

A total of 49,893,982 incidence records and 396,777,916 prescription records for 1,113,655 patients were recorded between January 2002 and December 2013. Among them, 225,406 patients met our defined criteria for a type 2 diabetes diagnosis (see Methods). After matching with controls on the basis of sex, age group, type of encounter, and diagnosis date, the number of cases (i.e., patients with type 2 diabetes) was reduced to 164,593. Among the cases, the female:male ratio was 50.3:49.7, and the highest proportion of type 2 diabetes diagnoses occurred during middle age (60.3%). The highest incidence was reported in 2002, and this was attributed to the extraction of the cohort based on the available insurance subscribers in 2002. Regarding the type of encounter, hospital outpatient was most frequent with 173,481 events, followed by hospital inpatient (n = 24,391). Regarding the average number of diagnoses per patient, cases had a significantly higher number of diagnoses, compared to controls (*p*-value < 0.001). In addition, the cases were diagnosed with a wider range of diseases (Table 1). The incidence diagnoses in the case group were ranged from 1 to 64,158 (64,158 patients had the same diagnosis). The most frequent diagnosis was “Dyspepsia” (K30), and its incidence rate was 38.98 per 100 patients with type 2 diabetes (Supplementary Dataset 1).

**Table 1:** Characteristics of the Study Population.

Variables	Cases (with type 2 diabetes) (n = 164,593)	Controls (without type 2 diabetes) (n = 164,593)	χ2	Chi-square *p*-value
	**Number (percent)**			
Sex			0	1
Male	81,749 (49.7)	81,749 (49.7)		
Female	82,844 (50.3)	82,844 (50.3)		
Age at first diagnosis			0	1
Middle age (40–59)	99,286 (60.3)	99,286 (60.3)		
Old age (60+)	65,307 (39.7)	65,307 (39.7)		
Year at first diagnosis			0	1
2002	25,082 (15.2)	25,082 (15.2)		
2003	14,580 (8.9)	14,580 (8.9)		
2004	13,345 (8.1)	13,345 (8.1)		
2005	14,515 (8.8)	14,515 (8.8)		
2006	11,240 (6.8)	11,240 (6.8)		
2007	11,075 (6.7)	11,075 (6.7)		
2008	12,247 (7.4)	12,247 (7.4)		
2009	11,625 (7.1)	11,625 (7.1)		
2010	11,352 (6.9)	11,352 (6.9)		
2011	13,768 (8.4)	13,768 (8.4)		
2012	12,602 (7.7)	12,602 (7.7)		
2013	13,162 (8)	13,162 (8)		
Type of encounters				
Outpatient in hospitals	141,458 (85.9)	141,458 (85.9)		
Inpatient in hospitals	19,863 (12.1)	19,863 (12.1)		
Outpatient in public health clinics	3,272 (2)	3,272 (2)		
Incidence records				
The avg. # of incidence per patient	33.8	18.3		*<0.001
The # of distinct diagnoses	1,373	1,324		

^*^p-value is based on the t-test.

### Type 2 diabetes progression patterns

A total of 1,373 distinct pairs (type 2 diabetes → D_1_) were identified from the full data set of type 2 diabetes patients. Of these, 833 pairs were considered significant using the cut-off points of a relative risk >1, *p*-value <0.001, and minimum occurrence count >10. Using the same criteria, we identified 1,233 significant trajectories of three diagnoses (type 2 diabetes → D_1_ → D_2_), and finally, 76,423 trajectories of four diagnoses (type 2 diabetes → D_1_ → D_2_ → D_3_). The full set of trajectories, including the number of patients, natural logarithm-scaled relative risks, and median and average durations, is listed in Supplementary Dataset 2.

To check the reliability of the trajectories, we counted the incidence and calculated the mean relative risks of common complications of type 2 diabetes. Among 76,423 trajectories, 19,486 contained at least one macro-, microvascular, or miscellaneous complication (Table 2). The mean number of relative risks of common type 2 diabetic complications in our trajectories exceeded 4. “Dyslipidemia (E78)” had the highest relative risk (relative risk = 21.19), whereas “Retinopathy and blindness (H28 and H36)” was the most frequent (count = 3,605).

**Table 2:** Common type 2 diabetic complications in the trajectories.

Description	ICD-10 code	Count	Mean duration, day(s)	Mean relative risk
**Macrovascular**				
Hypertension	I10-I15	2,146	804.02	12.40
Angina pectoris	I20	2,454	822.25	6.29
Myocardial infarction	I21	596	733.31	9.45
Atrial fibrillation	I48	516	736.70	7.20
Heart failure	I50	1,060	819.76	10.49
Cerebral infarction	I63	3,167	1,010.53	5.83
Stroke, not specified as haemorrhage or infarction	I64	256	694.14	4.84
Occlusion and stenosis of precerebral arteries, not resulting in cerebral infarction	I65	268	844.06	7.53
Occlusion and stenosis of cerebral arteries, not resulting in cerebral infarction	I66	178	709.33	4.25
Atherosclerosis	I70	823	888.73	12.01
Other peripheral vascular diseases	I73	2,089	1,051.65	5.96
**Microvascular**				
Renal failure (nephropathy)	N17, N18, N19	2,086	866.66	10.51
Retinopathy and blindness	H28, H36	3,605	852.95	7.65
Neuropathy	G59, G63	2,617	934.30	8.26
Foot ulcers and amputation	L97	68	797.90	36.29
**Miscellaneous**				
Dyslipidemia	E78	388	784.06	21.19
Gastroparesis	K31	324	828.47	20.27
Viral Hepatitis	B16-B18	500	756.38	8.59
Hearing impairment	H90-H95	2,971	828.61	8.51

**Figure 1:**
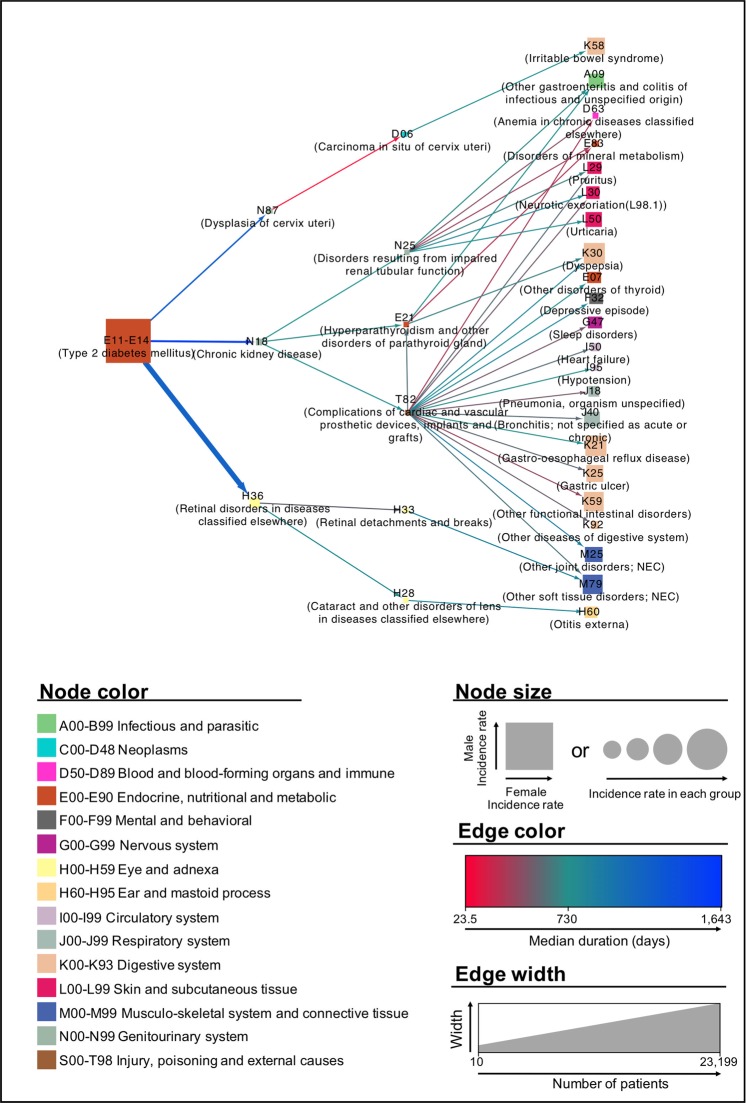
The top 30 trajectories with the highest average relative risks in a full population dataset analysis. The trajectories included microvascular, macrovascular, and miscellaneous complications.

We included the top 30 trajectories with the highest average relative risks in our comprehensive visual overview of the progression patterns (Fig. 1). In these trajectories, the mean interval from the type 2 diabetes diagnosis to the second diagnosis (D_1_) was relatively long (936 days), compared to the mean intervals from the second to the third diagnosis (D_2_) and from the third to the fourth diagnosis (D_3_) (571.95 and 560.72 days, respectively). In most trajectories, the second diagnoses mostly involved type 2 diabetes-associated diseases, such as chronic kidney disease^22–25^, retinal disorders^26–28^, and dysplasia of the cervix uteri^29–32^. “Complications of cardiac and vascular prosthetic devices, implants and grafts” (T82) were the most frequent out-degrees among the third diagnoses (18), and these served as bridges to many of the fourth diagnoses.

### Patterns of progression by sex and age

**Figure 2:**
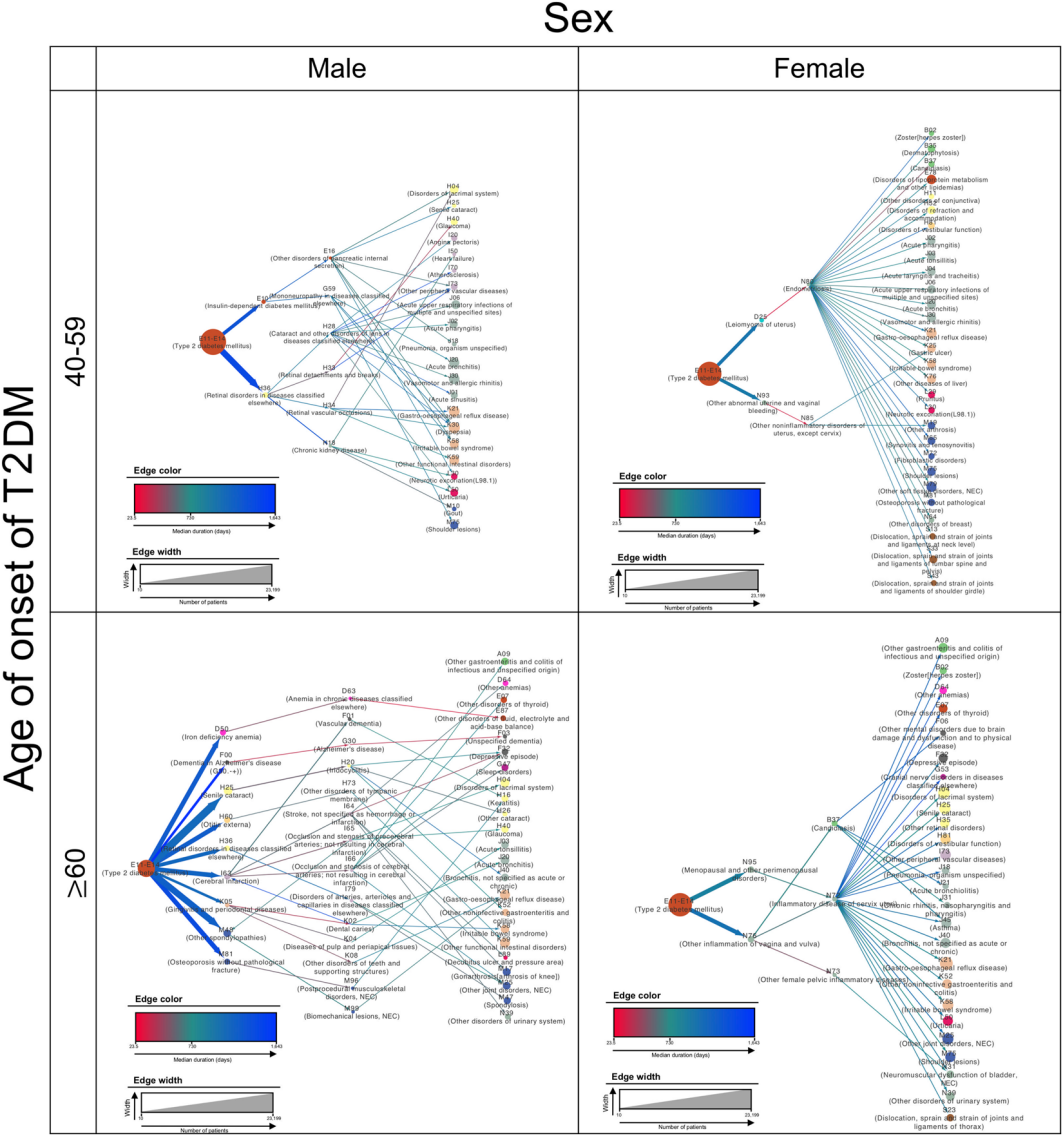
The top 30 trajectories with the highest average relative risks in analyses of 4 different age-sex groups. The trajectories of each group exhibited distinctive characteristics.

We further divided the full data set of patients with type 2 diabetes into four groups to investigate age- and sex-related differences in progression patterns and extracted the significant trajectories in each group. A total of 5,137, 4,021, 3,700, and 5,152 significant trajectories with four diagnoses were extracted for the female middle-aged, male middle-aged, female older-aged, and male older-aged groups, respectively (Supplementary Dataset 2). The top 30 trajectories with the highest average relative risks for each group are visualized in Fig. 2.

A comparison of the top 30 trajectories by sex revealed a clear difference in the progression patterns between the male and female groups. For example, the female groups yielded few diagnoses in the second and third levels (2 and 2, respectively, for the middle-aged group and 2 and 3, respectively, for the older-aged group), of which most were classified as “Diseases of the genitourinary system (N00–N99)”. In contrast, the male groups yielded a relatively large number of various categorized diagnoses in the second and third levels (2 and 6, respectively, for the middle-aged group and 9 and 14, respectively, for the older-aged group). Moreover, some specific diagnoses had an exceedingly high out-degree in the female groups; these included “Endometriosis (N80)”, with 28 out-degrees in the middle-aged female group, and “Inflammatory disease of cervix uteri (N72)”, with 23 out-degrees in the older-age female group.

## Discussion

In this study of a population-wide claims dataset, we investigated the characteristics of temporal links, including the relative risks, occurrence counts and durations, identified between type 2 diabetes and accompanying comorbidities, and constructed and visualized the temporal progression patterns of type 2 diabetes in terms of trajectory interpretation. Additionally, we constructed trajectories based on combinations of age and sex and comparatively analyzed the differences in progression among the indicated subgroups. Notably, our trajectories not only comprised the well-known examples such as “Type 2 diabetes (E11-E14)” → “Retinal disorders in diseases classified elsewhere (H36)” → “Polyneuropathy in diseases classified elsewhere (I73)” → “Other peripheral vascular diseases (I73)” but also included more recently studied trajectories such as “Type 2 diabetes (E11-E14)” → “Depressive episode (F32)” → “Postprocedural musculoskeletal disorders, NEC (M96)” → “Other disorders of thyroid (E07)”^33–35^. We further identified age- and sex-related differences in trajectories. Specifically, among the top 30 trajectories with the highest average relative risks, those of female groups contained high-degree nodes, while those of male groups included various types of diagnoses at the 3^rd^ and 4^th^ levels. Additionally, older-age males had the widest variety of diagnoses. In all subgroups, the interval between a type 2 diabetes diagnosis and a 2^nd^ level diagnosis was longer than the intervals between other levels.

Our results were consistent with the findings from previously published studies. As mentioned in the Introduction, the complications of type 2 diabetes can be largely classified as macrovascular, microvascular, and miscellaneous. The trajectories of type 2 diabetes identified in our study also included all macrovascular (peripheral vascular diseases, stroke and acute myocardial infarction) and microvascular complications (glomerular disorders and mononeuropathies or polyneuropathies), as well as other representative complications (depressive episode and thyroid disorders). Moreover, our trajectories revealed several connections between microvascular and macrovascular complications, thus suggesting that these complications can exist either on a continuum or as discrete entities^11^.

Interestingly, our trajectories included several complications that have not been previously identified or considered as common manifestations in patients with type 2 diabetes. For example, several trajectories included viral hepatitis (ICD-10 codes: B16, B17 and B18). Several studies have already provided evidence that may suggest a relationship between viral hepatitis and type 2 diabetes. Impaired immunity has been well demonstrated in diabetics, and the prevalence of viral hepatitis infection is relatively frequent among patients with immune suppression^36–39^. However, to seek the reason why many of our trajectories consisted of viral hepatitis, we must consider the cause underlying the significant relationship between these disease entities in our trajectories^40^. Specifically, our dataset was derived from the Korean population, which has a high prevalence of viral hepatitis^41^. This observation suggests that the trajectories of this study include subject-specific characteristics, which may limit the generalizability of our findings to other population. However, this information may also be a strength, given its potential use for population-specific guidelines. Thyroid disorder is another example of a less well-known complication of type 2 diabetes. Our findings are supported by those of Hage *et al*., who reported that diabetes and thyroid disorders, both of which involve endocrine system dysfunction, tended to coexist in patients, and of Sotak *et al*., who stated that patients with type 2 diabetes had a higher prevalence of hyperthyroidism and autoimmune thyroid disease. Our and previous findings thus validate the temporal relationship between type 2 diabetes and thyroid disease^42,43^.

Despite the potential usefulness of these findings, a few limitations must be considered. The suitability of claims data for clinical research use is limited, given the lack of diagnostic details encoded by the ICD coding system. For example, Latent Autoimmune Diabetes of Adulthood (LADA) on insulin and/or oral glucose lowering agents were not able to be excluded from our cohort since there is no formal consensus regarding clear diagnostic criteria. Moreover, diagnoses may be over-diagnosed or misdiagnosed to meet insurance coverage criteria. Accordingly, it would be difficult to ensure that the patient actually presented with the diagnosed diseases. We must also consider that the initial date of physician diagnosis, which we considered to be the incidence date, may be biased given the lack of narrative text in claims data to indicate when and for what duration the patients had experienced symptoms. For instance, the trajectories of middle-aged men exhibited a pathologically inexplicable progression from type 2 diabetes to insulin-dependent diabetes (Fig. 1). This comorbid pair was also identified in previous trajectories constructed based on Danish claims data, suggesting the initial misdiagnosis of insulin-dependent diabetes as type 2 diabetes^21^. Considering these limitations, our category of “accompanying comorbidities” may be more precisely defined as “accompanying diagnoses”.

We further note that although we adjusted for potential confounders, including sex, age, encounter type, and date of diagnosis, we did not consider other potentially important confounding variables that may have affected disease progression, such as prescription and treatment information, smoking habits, and the general health status.

Despite these limitations, the type 2 diabetes trajectories presented in this report may improve patient outcomes by facilitating early disease recognition. Even though we cannot determine the exact timing of type 2 diabetes-related complications simply based on the claims data, our trajectories could indicate the relative temporal order of complications after a diagnosis of type 2 diabetes. Additionally, sex- and age-specific trajectories could serve as useful tools that would help clinicians determine when and which prescriptions and treatments should be administered to patients with type 2 diabetes. These trajectories could also reveal different pre-disposing factors by providing information about the types and time courses of the diagnoses expected to occur after a type 2 diabetes diagnosis, or about the onset of complications in specific sex and age groups within a relatively short time period. Although many previous studies investigated the development of complications in patients with type 2 diabetes using competing risk analyses, these were limited to the outcomes of a few well-known complications^44,45^. Only considering the development of well-known type 2 diabetic complications, our study may provide less information than previous studies. However, as we aimed to construct a map of type 2 diabetes that would not only allow us to explore the development of well-known complications but also discover and reveal previously unknown relationships, we focused more on the relationships between type 2 diabetes and all possible accompanying comorbidities, rather than specifically defining and confirming the causal relationships between type 2 diabetes and well-known complications.

In future studies, we aim to explore the patterns of prescription for drug repositioning to determine whether specific drugs can either cause or prevent the comorbidities that accompany type 2 diabetes, based on the networks constructed in this study.

## Methods

### Data source

The National Health Insurance Service (NHIS) is a universal health insurance system that covers approximately 98% of the entire 50.6 million South Korean population. For this study, we used the National Health Insurance Service–National Sample Cohort (NHIS-NSC), which initially included 2.2% of the total eligible Korean population (approximately 1 million medical insurance subscribers) in 2002 and followed every hospital visit of these subjects for 11 years (2002–2013), regardless of the type of encounter^46^. The NHIS-NSC is a relational database comprising 118 variables, including personal demographics such as age and sex, treatment, disease types, and prescriptions. To compensate for annual losses due to participant deaths or disqualification from health services, newborns were sampled using the 2.2% sampling rate and added to the cohort each year between 2003 and 2013. The NHIS-NSC has been used extensively in numerous publications over recent years and has proved its reliability and validity^47–50^.

The diagnostic codes used in this study are documented in the Korean Classification of Diseases, 6th version (KCD-6), a modified version of the International Classification of Diseases (ICD-10). These classifications mainly differ only in the use of the 5^th^ character, which indicates anatomic sites, and the designation of codes U00–U99 to aspects of Korean medicine in the KCD-6. We refined the diagnosis codes using the first three-digit codes, which commonly designate the main category in both classification systems. Codes corresponding to “Pregnancy, childbirth and the puerperium (O00–O99),” “Symptoms, signs and abnormal clinical and laboratory findings, NEC (R00–R99),” “Codes for special purposes (U00–U99),” “External causes of morbidity and mortality (V01–Y98),” and “Factors influencing health status and contact with health services (Z00–Z99)” were excluded from the study dataset because they do not indicate specific disease. To exclude repeated admissions for the same diagnosis, we only used the incidence records corresponding to each diagnosis in each patient.

This study was approved by the Ajou University Hospital Institutional Review Board [AJIRB-MED-EXP-18-293], which waived the requirement for informed consent. The study complied with the tenets of the Declaration of Helsinki.

### Diagnostic criteria for type 2 diabetes

We narrowed our sample to patients aged ≥40 years, as type 2 diabetes is most frequently diagnosed in this age group. Type 2 diabetes was defined as either ≥2 visits for which type 2 diabetes-related diagnostic codes (E11 [Type 2 diabetes], E12 [Malnutrition-related diabetes mellitus], E13 [Other specified diabetes mellitus], and E14 [Unspecified diabetes mellitus]) were assigned between 2002 and 2013 or 1 visit with a type 2 diabetes diagnosis and a filled prescription for diabetes-related medications, including metformin, nateglinide, repaglinide, insulin, sitagliptin, saxagliptin, linagliptin, alogliptin, acarbose, glimepiride, glibenclamide, gliclazide, glipizide, rosiglitazone, pioglitazone, dapagliflozin, ertugliflozin, liraglutide, exenatide, and dulaglutide. This definition of type 2 diabetes was adopted from Yul *et al*.^51^.

### Extraction of significant type 2 diagnosis-accompanying comorbidity pairs

**Figure 3:**
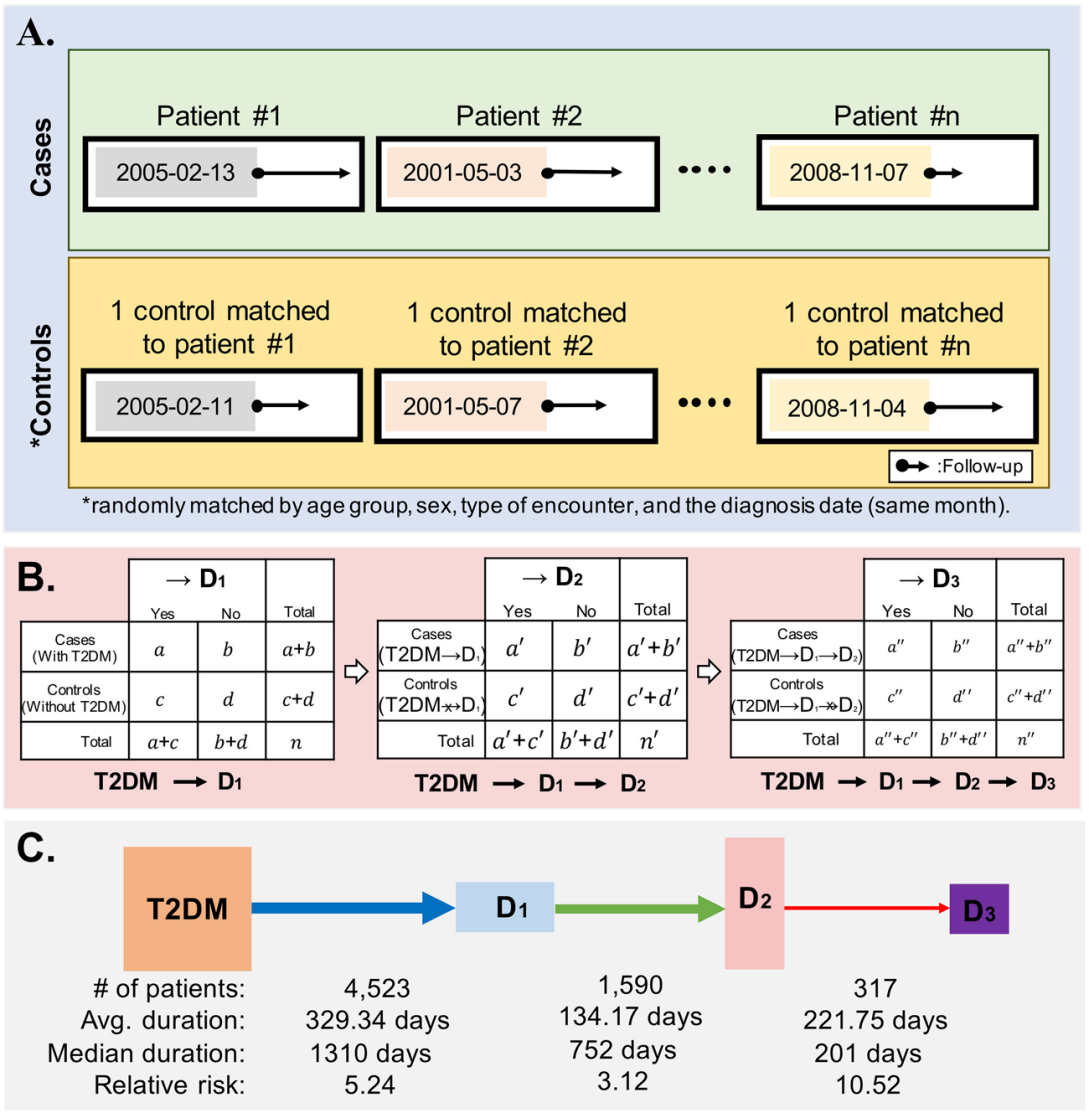
Procedure used to construct type 2 diabetes trajectories. (**A**) Each case was matched to a randomly selected control according to age group, sex, type of encounter, and diagnosis date. (**B**) Cases and controls were newly defined, and Fisher’s exact test was applied to each step until the trajectories included four diagnoses. (**C**) The number of occurrences, average duration, median duration, and relative risk were calculated for each links.

We subjected patients with type 2 diabetes to a case-control study to identify the relationships between type 2 diabetes and accompanying comorbidities. Each diagnosed patient was matched in a one-to-one ratio with a randomly selected patient who had never been diagnosed with type 2 diabetes but was matched in terms of age group (at 5-year intervals), sex, and type of hospital encounter during the same month, without replacement (Fig. 3A). The type of hospital encounter included hospital inpatient, hospital outpatient, and public health clinic outpatient, and this variable was matched between the case and control groups was to minimize the confounding effects of baseline bias. The month of diagnosis was also matched to exclude the possibility of a change in the diagnostic method and the potential effects of seasonal differences. After defining the cases and appropriate controls, we extracted all possible combinations (or pairs) of type 2 diabetes and accompanying comorbidities from the diagnostic records of patients with type 2 diabetes in the case group. The incidence of each pair was counted and recorded. To access the statistical significance of the pairs, we generated a 2 × 2 contingency table for each pair and used the relative risk to measure the strength of each association, as shown in Fig. 3B. The relative risk estimates and associated *p*-values were calculated using Fisher’s exact test with the Bonferroni correction. For example, the relative risk of progression from type 2 diabetes to D_1_ was calculated as follows:

$$R{R}_{type2diabetesmellitus\to {D}_{1}}=\frac{a/(a+b)}{c/(c+d)}$$

where $a$ is the number of patients diagnosed with D_1_ after the diagnosis of type 2 diabetes; $b$ is the number of patients never been diagnosed with D_1_ after the diagnosis of type 2 diabetes; $c$ is the number of patients never been diagnosed with type 2 diabetes but previously diagnosed with D_1_; and $d$ is the number of patients diagnosed with neither disease. A corrected *p*-value < 0.001 and a relative risk >1 were considered significant. We eliminated any associations of between type 2 diabetes and other diagnoses with fewer than 10 event counts to address the bias associated with a small sample size.

### Diagnostic trajectories

We defined the association of type 2 diabetes → D_1_ by comparing the groups with and without type 2 diabetes. Subsequently, we newly defined the case and control group as the groups with and without D_1_ after the diagnosis of type 2 diabetes, respectively, and used these groups to test the significance of the type 2 diabetes → D_1_ → D_2_ trajectory. During this process, the same variables (age group, sex, type of encounter, and diagnosis date) were used to match the cases and controls, and Fisher’s exact test was applied using the same *p*-value, relative risk, and minimum count cut-offs throughout the analysis (Fig. 3B).

To extracting the trajectories of type 2 diabetes → D_1_ → D_2_ → D_3_, we selected a group of patients who were diagnosed in the order of D_1_ and D_2_ after a type 2 diabetes diagnosis as the case group and a group that had been diagnosed with D1 but not D_2_ after a type 2 diabetes diagnosis as the control group. All matching processes and statistical tests were applied as described above (Fig. 3B). Finally, we calculated the average and median duration (in days) of each link of the four long trajectories (Fig. 3C).

### Visualization of the diagnostic trajectories

To easily identify the overall pattern of type 2 diabetes progression, we depicted the four long trajectories as nodes (i.e., diagnoses) with directed and weighted edges. The color of each node corresponds to the ICD-10 disease category, and the shape of each node is indicated by a rectangle to indicate showing sex-related differences; the rectangle width is proportional to the number of female cases, while the rectangle height is proportional to the number of male cases. The edge colors represent duration, with red and blue shades indicating shorter (<730 days) and longer durations (>730 days), respectively. An edge corresponding to a duration of exactly 730 days (2 years) is indicated in green. The edge thickness is proportional to the count of each link. Moreover, only the top 30 trajectories in terms of average relative risks were visualized to reduce complexity and ensure effective comprehension and visual exploration. The relative risks were scaled logarithmically to respond to skewness toward large values.

To investigate the effects of sex and age of type 2 diabetes onset, we constructed graphs after dividing patients into four groups by sex and age: males aged 40–59 years (i.e., middle-aged), females aged 40–59 years, males aged >60 years (i.e., older-aged), and females aged >60 years. Because the sex was fixed in each group, the nodes were modified from a rectangular to a circular shape. The node and edge attributes were reset according to the individual group characteristics rather than the full population data.

## Supplementary information

Supplementary information.

Supplementary dataset 1.

Supplementary dataset 2.

## Data Availability

The NHIS-NSC dataset can only be accessed after approval by the NHIS and it cannot be shared publicly due to data sharing policy. Further information on the dataset can be found at https://nhiss.nhis.or.kr/bd/ab/bdaba021eng.do.
