# Supplementary material for: Temporal trajectories of accompanying comorbidities in patients with type 2 diabetes: a Korean nationwide observational study
Source: Sci Rep. 2020 Mar 26;10:5535. doi: 10.1038/s41598-020-62482-1 (PMC7099011; doi:10.1038/s41598-020-62482-1)
Supplement: Supplementary file 1 — Supplementary information. [file 41598_2020_62482_MOESM1_ESM.pdf]

Temporal trajectories of accompanying comorbidities in patients with type 2 diabetes: a Korean nationwide observational study

Eugene Jeong[1], Namgi Park[2,3], Yujeong Kim[2], Ja Young Jeon[4], Wou Young Chung[5], Dukyong Yoon[2,3*]

1. Department of Biomedical Informatics, School of Medicine, Vanderbilt University, Nashville, TN, USA

2. Department of Biomedical Informatics, Ajou University School of Medicine, Suwon, Gyeonggi-do, Republic of Korea

3. Department of Biomedical Sciences, Ajou University Graduate School of Medicine, Suwon, Gyeonggi-do, Republic of Korea

4. Department of Endocrinology and Metabolism, Ajou University School of Medicine, Suwon, Gyeonggi-do, Republic of Korea

5. Department of Pulmonology and Critical Care Medicine, Ajou University School of Medicine, Suwon, Gyeonggi-do, Republic of Korea

*Corresponding Author: Dukyong Yoon (E-mail:d.yoon.ajou@gmail.com)

Supplementary dataset 1. Incidence of each diagnoses according to KCD-6 code in case and control.

Supplementary dataset 2. The full set of significant trajectories including the number of patients (count), average and median durations, and natural logarithm-scaled relative risks for total, male middle-aged (age between 40 and 60), female middle-aged, male older-aged (age over 60), and female older-aged groups.
